# Intelligent Compaction System for Soil-Rock Mixture Subgrades: Real-Time Moisture-CMV Fusion Control and Embedded Edge Computing

**DOI:** 10.3390/s25175491

**Published:** 2025-09-03

**Authors:** Meisheng Shi, Shen Zuo, Jin Li, Junwei Bi, Qingluan Li, Menghan Zhang

**Affiliations:** 1 School of Civil Engineering, Shandong Jiaotong University, 5 Jiaoxiao Road, Jinan 250357, China; 23107030@stu.sdjtu.edu.cn (M.S.);; 2 Department of Geotechnical Engineering, School of Civil Engineering, Southwest Jiaotong University, No. 111, North Section 1, Second Ring Road, Chengdu 610031, China

**Keywords:** intelligent compaction, mixed earth and stone road base, water content of roadbed, near-infrared spectrum

## Abstract

The compaction quality of soil–rock mixture (SRM) subgrades critically influences infrastructure stability, but conventional settlement difference methods exhibit high spatial sampling bias (error > 15% in heterogeneous zones) and fail to characterize the overall compaction quality. These limitations lead to under-compaction (porosity > 25%) or over-compaction (aggregate fragmentation rate > 40%), highlighting the need for real-time monitoring. This study develops an intelligent compaction system integrating (1) vibration acceleration sensors (PCB 356A16, ±50 g range) for compaction meter value (CMV) acquisition; (2) near-infrared (NIR) moisture meters (NDC CM710E, 1300–2500 nm wavelength) for real-time moisture monitoring (sampling rate 10 Hz); and (3) an embedded edge-computing module (NVIDIA Jetson Nano) for Python-based data fusion (FFT harmonic analysis + moisture correction) with 50 ms processing latency. Field validation on Linlin Expressway shows that the system meets JTG 3430-2020 standards, with the compaction qualification rate reaching 98% (vs. 82% for conventional methods) and 97.6% anomaly detection accuracy. This is the first system integrating NIR moisture correction (R^2^ = 0.96 vs. oven-drying) with CMV harmonic analysis, reducing measurement error by 40% compared to conventional ICT (Bomag ECO Plus). It provides a digital solution for SRM subgrade quality control, enhancing construction efficiency and durability.

## 1. Introduction

Soil–rock mixture (SRM) subgrades utilize fills comprising cohesive/sandy soils blended with rock aggregates (crushed stone, boulders, or gravel). These engineered materials integrate the advantageous geotechnical properties of both constituents, exhibiting superior deformation resistance and drainage capacity. However, significant spatial heterogeneity in rock content (coefficient of variation: 15–30% in field investigations [1]) and compaction density (differences up to 12% within 10 m^2^ [2]) poses critical challenges: under-compaction induces high porosity (>25%) and post-construction settlement exceeding 300 mm [3], while over-compaction fractures aggregates (fragmentation rate > 40% for rock content > 60%) and degrades the load-bearing skeleton structure.

Mechanistically, SRM compaction fundamentally differs from homogeneous soil or rockfill compaction. Early research by Xie et al. established experimental frameworks for gradation-dependent SRM compaction through large-scale testing [4], but their studies ignored the influence of moisture content on compaction efficiency. Although subsequent research established standardised laboratory compaction procedures, it failed to resolve the issue of real-time calibration of field compaction indicators under varying moisture content conditions—a critical gap for heterogeneous SRM. Ji et al. advanced micro-mechanical understanding via Discrete Element Method (DEM) simulations, analyzing particle-scale contact forces and displacement evolution during compaction [5].

Technological progress has enabled roller-integrated compaction monitoring. Pioneered by Heinz’s Intelligent Compaction Technology (ICT) [6], Continuous Compaction Control (CCC) systems [7,8,9,10] employ vibratory drum-mounted sensors to acquire dynamic response signals. However, in SRM applications, these systems exhibit two critical limitations: (1) CMV indices are biased by 8–15% in high-moisture conditions (>10%) due to fine particle lubrication [11], and (2) they fail to account for rock content heterogeneity, leading to the misjudgment of compaction quality in stone-rich zones (>50% rock content) [12]. Sandström further refined vibratory roller performance characterization [13,14,15,16].

Despite these advances, conventional ICT exhibits significant limitations in SRM applications [17,18]. A core issue is measurement validity—whether derived indices accurately reflect compaction quality—stemming partly from the inherent constraints of compaction meter value (CMV) methodologies. This challenge is exacerbated in SRM subgrades, where polydisperse fills form metastable dense-suspension structures that introduce substantial mechanistic uncertainty. Conventional methods have a high spatial sampling bias, moisture-induced CMV errors (8–15% in high moisture), and a lack of real-time feedback for heterogeneous SRM.

To address the inability of existing ICT to provide real-time, intuitive compaction quality feedback, this study integrates field observations with laboratory experimentation. We propose a dual-mode CMV control framework with two key innovations: (i) real-time moisture correction via near-infrared spectroscopy (NIR) with a calibration model (R^2^ = 0.96 vs. oven-drying method) to eliminate moisture-induced bias [19,20]; (ii) vibration harmonic analysis (extracting 2nd–4th harmonics via FFT) to characterize aggregate skeleton formation, which differs from conventional ICT’s single-frequency analysis [7]; and (iii) an embedded edge computing system based on Python (50 ms latency) to achieve real-time data fusion and realize full-process monitoring. Field tests have shown a pass rate of 98% and an anomaly detection accuracy rate of 97.6%. Mobile edge computing (MEC) has been identified as one of the promising technologies able to improve the computing capabilities of wireless devices [21,22,23]. Integration with centimeter-level GNSS trajectory analysis [24,25,26,27,28] generates spatially resolved moisture-CMV distribution cloud maps, enabling targeted compaction remediation in under-compacted zones.

Implementation on the Linlin Expressway (Section 4) testbed achieved a 97.6% anomaly detection accuracy (vs. 72.3% for manual inspection [17]) and elevated the SRM compaction compliance to 98% (from 82% with conventional ICT), demonstrating its superiority in complex SRM scenarios. This methodology provides both theoretical underpinnings and practical tools for the quality control of complex subgrade compaction.

## 2. Research on Compaction Characteristics of Soil and Gravel Mixtures

### 2.1. Earth and Rock Mix Take

In the southwestern plains of Shandong Province, the Linlin Expressway’s fourth-section multi-point soil sampling area is characterized by lithologies primarily from the Taishan Group’s Taipingding Formation and Yanyingguan Formation, including diorite, dioritic hornblende granite gneiss (migmatite), Yanshanian intrusive diorite, and their weathered materials.

### 2.2. Particle Analysis of Soil and Gravel Mixtures

To evaluate the gradation composition rationality, soil–rock mixtures from discrete sampling sites were subjected to particle-size analysis. Field samples (W-1 to W-4) were processed by removing aggregates > 20 mm (per JTG 3430-2020 [29] for laboratory test specimen preparation) to prepare specimens. Air-dried mixtures were quartered (to ensure representative sub-samples) and fractionated through a 2 mm sieve, separating the <2 mm soil matrix and >2 mm rock aggregates for sieve analysis. The sub-2 mm fraction (soil matrix) and super-2 mm fraction (rock aggregates) underwent fine and coarse sieve analyses, respectively. The mass of materials retained on each sieve was quantified.

**Figure 1:**
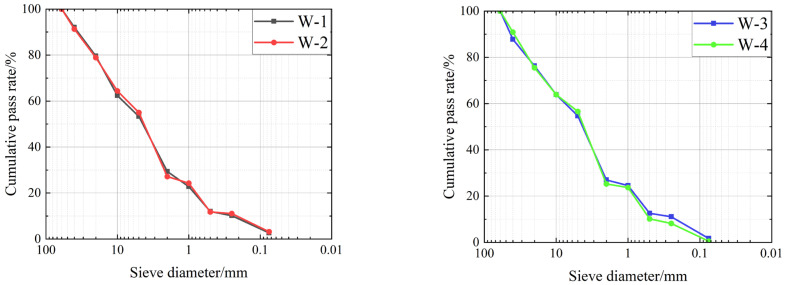
Grading curves.

Based on the results of the particle analyses, the gradation composition of the soil–rock mixtures at several sampling points was determined and the particle size distribution curves were plotted. This is shown in Figure 1 (*n* = 3 replicates, error less than 5%). The curves for (a) W-1 and W-2, compared to (b) W-3 and W-4, show finer grading (more particles smaller than 5 mm), while W-3 and W-4 contain more coarse aggregates (>10 mm). Sieve diameters are in millimeters (mm).

The uniformity coefficient (*C*_u_) and curvature coefficient (*C_c_*) at each sampling location were calculated from the particle-size distribution curve. Soil–gravel mixtures retained on the 60 mm sieve were excluded from the gradation analysis, as specified in Table 1:

According to ‘Highway Geotechnical Test Specification’ (JTG 3430-2020), soil–rock mixtures with a soil mass significantly less than the stone mass and a fine-grained soil proportion of less than 5% of the total mass are classified as well-graded. The specification defines well-graded soils as those with a uniformity coefficient (*C*_u_) ≥ 5 and a curvature coefficient (*C_c_*) of 1–3. Notably, none of the four locations met these criteria (*C*_u_ ≥ 5, *C_c_* = 1–3), classifying them as poorly graded. Specifically, W-3 exhibits the highest *C*_u_ (40.34) but lowest *C_c_* (0.79), indicating a lack of intermediate particles, which exacerbates segregation during paving. Poor gradation increases the risk of uneven compaction (density variation > 5%) and post-construction settlement, as fine particles fail to fill voids between coarse aggregates.

### 2.3. Indoor Compaction Tests of Soil and Gravel Mixtures

The engineering physical properties of soil–rock mixture-filled subgrades exhibit complex behaviors, influenced not only by particle gradation and vibration-induced rock fragmentation but also by the stone content and optimum moisture content. Among these factors, the degree of compaction in soil–rock mixed subgrades is critical, as it directly affects the subgrade’s load-bearing capacity and post-construction settlement deformation. In practical engineering, compaction quality inspections of soil–rock mixed subgrades often report values exceeding 100%, complicating on-site quality assessment. Laboratory tests were conducted using both the manual light compaction method and surface vibration compaction testing.

#### 2.3.1. Preparation of Soil and Gravel Mix Compaction Test Specimens

Soil–rock compaction specimens were prepared based on particle gradation data derived from sieve analysis. To meet ASTM D698 (manual light compaction) and ASTM D4253 (surface vibration compaction) requirements, coarse aggregates > 20 mm were crushed to 5–20 mm using a jaw crusher, with gradation controlled by sieving (20 mm, 10 mm, 5 mm sieves). The quadratic mixing method was employed: (1) pre-mix stones and soil in dry state for 5 min; (2) add the target moisture content and mix for another 10 min to ensure homogeneity, verified by random sampling (moisture variation < 0.5%).

Specimens were oven-dried at 105 °C for 10 h and prepared at six stone content ratios (20%, 30%, 40%, 50%, 60%, 70% by mass). For each stone content, six sub-specimens were configured at varying moisture contents (7%, 8%, 9%, 10%, 11%, 12%) following the quadratic mixing method. Upon reaching target moisture levels, mixtures were homogenized and sealed in black polyethylene bags for a minimum of 12 h to ensure moisture equilibrium.

#### 2.3.2. Results and Analyses

**Figure 2:**
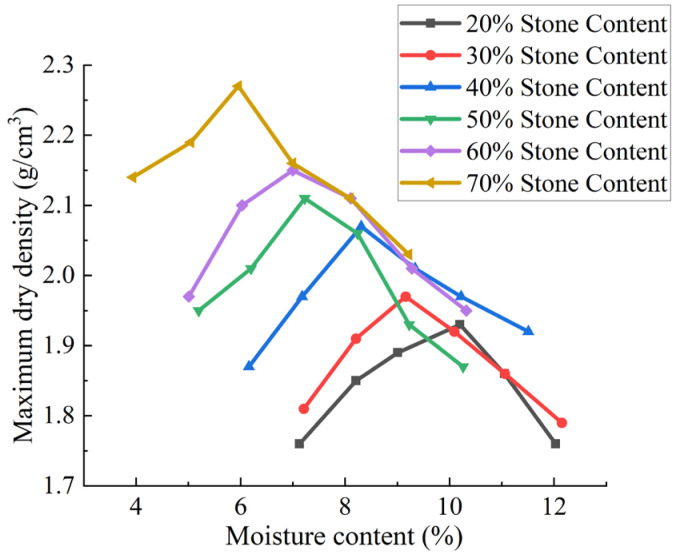
Optimum moisture content curve of manual compactor method.

**Figure 3:**
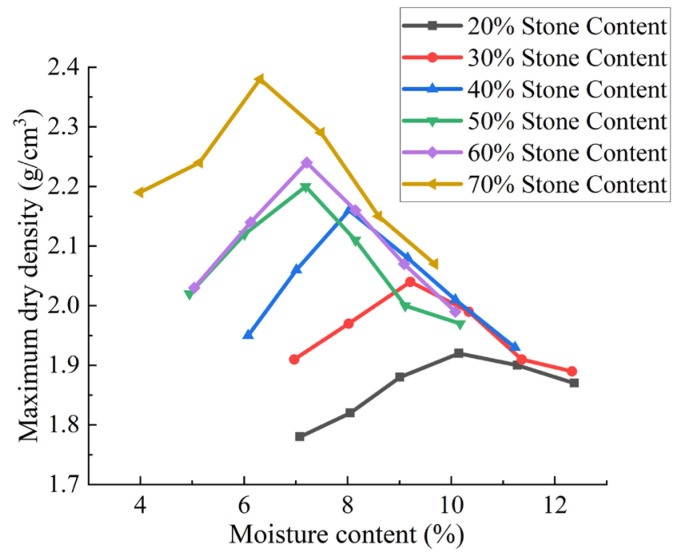
Optimum moisture content curve of surface vibration compactor method.

The water content was measured via oven-drying (105 °C, 24 h) on three cores (diameter 50 mm) from each compacted specimen. The optimum moisture content curves for manual light compaction and surface vibration compaction are shown in Figure 2 and Figure 3, respectively. The moisture content data represent the means of three replicates (SD < 0.2%)

As shown in Figure 2 and Figure 3, the compaction curves of soil–stone mixtures are similar to those of fine-grained soils (homogeneous soils), characterized by an increase in dry density with increasing moisture content, followed by a peak. The maximum dry density of soil–stone mixtures increases with increasing stone content, but the optimal moisture content shows a decreasing trend.

For the two test methods, the coefficient of variation (*C·V*) of the maximum dry density was calculated for different rock contents and moisture contents relative to the compacted maximum dry density. The coefficient of variation (*C·V*) was calculated to evaluate data stability using Equation (1):

(1) C·V=(SD/MN)×100%

where *SD* = standard deviation of maximum dry density (*MDD*) across three replicates; *MN* = mean *MDD*. A higher *C·V* (>10%) for stone content > 60% indicates increased test variability due to aggregate fragmentation.

Calculations indicate that the coefficient of variation (*C·V*) of the *MDD* increases with the stone content in soil–rock mixtures, while the *MDD* itself rises with increasing stone content and the optimum moisture content decreases. In conventional construction, soil–rock mixtures often exhibit significant particle heterogeneity, leading to material segregation and gradation inconsistency. This results in the formation of “skeletal void” structures (where large stones are not filled with fine soil) in gravel-rich zones, contrasting with fine soil-dominated areas. The moisture content in fine soil zones is typically higher than in gravel-rich zones, causing localized softening during compaction.

Laboratory tests reveal that soil–rock mixtures with varying particle gradations exhibit non-uniform mechanical properties. The inadequate control of soil–stone proportions or insufficient mixing during construction can lead to delamination or segregation after paving, forming skeletal voids between large stones where fine soil fails to fully fill, resulting in localized densification defects. Laboratory tests reveal that stone content > 60% leads to ‘skeletal void structures’—large aggregates form a framework with insufficient fine particles to fill voids (porosity > 20%), as observed in W-3 specimens. Conversely, fine soil-dominated zones (stone content < 30%) exhibit moisture-induced softening when the water content exceeds 12%, reducing the bearing capacity by 15%. These findings guide field compaction: target stone content 30–50% and moisture within 7–10% to balance density and stability.

## 3. Intelligent Compaction System Construction

An intelligent compaction system, as an emerging technology for enhancing subgrade construction quality and efficiency, is increasingly adopted in highway projects in recent years. While it offers the advantages of real-time monitoring and data recording—unmatched by traditional compaction methods—it struggles to provide reliable construction guidance for mixed soils with diverse moisture contents, plasticity indices, and distinct mechanical responses during compaction. This study presents a novel intelligent compaction system tailored for heterogeneous subgrades, such as soil–rock mixed-fill structures.

### 3.1. Intelligent Vibratory Roller

#### 3.1.1. Intelligent Vibratory Roller Structure Composition

An intelligent vibratory roller (model: XCMG XS263J) with real-time detection capabilities was modified from a conventional roller (drum diameter: 1.6 m, width: 2.1 m, maximum excitation force: 400 kN) by integrating multiple sensor systems. Its core components include (1) a vibratory roller mechanism with adjustable frequency (20–30 Hz) and amplitude (0.8–1.5 mm); (2) a real-time monitoring system (sampling rate: 1 kHz) for compaction indices (CMV, moisture); (3) an intelligent guidance program that triggers alerts when CMV deviates from the target range (15–25 kN·s/m) or moisture exceeds 10%; and (4) a fault diagnosis module (response time < 1 s) for sensor malfunctions. This configuration enables the full-process control and feedback of the roller’s operations, thereby facilitating the data-driven optimization of subsequent construction procedures.

#### 3.1.2. Structural Composition of Intelligent Compaction System of New Intelligent Vibratory Roller

**Figure 4:**
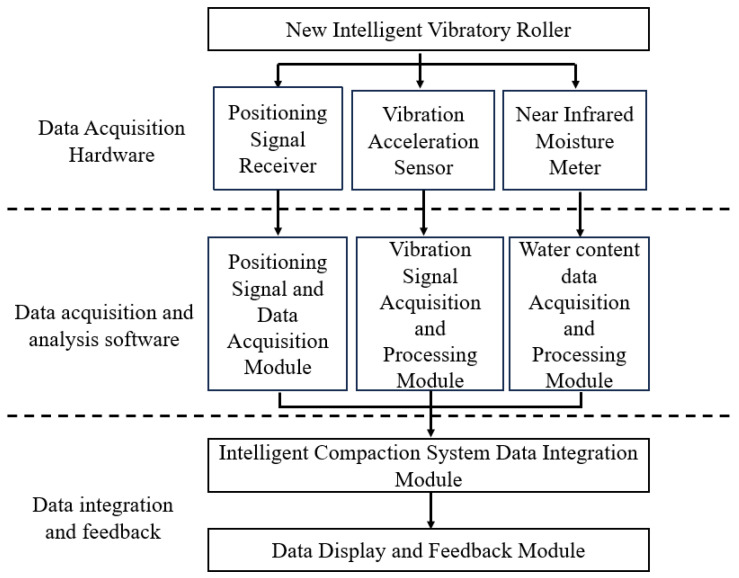
Main structural components of the intelligent compaction system of the new intelligent vibratory roller.

The main components of the new vibratory roller intelligent compaction system are shown in Figure 4 below. The system shown in the figure integrates real-time sensors (left module) for humidity and density monitoring, and the data is transmitted to the central processing unit (middle module) for immediate analysis.

As shown in Figure 4, the advantages of this system include the automatic adjustment of the drum frequency based on feedback signals (right module), which reduces manual intervention compared to traditional systems and achieves higher compaction uniformity.

Each module includes specific parameters: Vibration Signal Acquisition Module (sampling rate: 2000 Hz, 16-bit resolution); Moisture Data Acquisition Module (NIR spectrometer, 1300–2500 nm wavelength); and Positioning Module (GNSS RTK, horizontal accuracy: ±2 cm). Data transmission between modules uses Ethernet (100 Mbps) for low latency.

#### 3.1.3. Roadbed Intelligent Compaction System Main Equipment Composition

**Figure 5:**
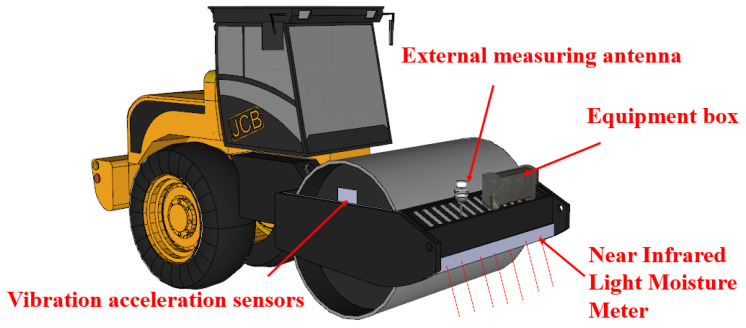
Intelligent compaction system equipment composition diagram.

The intelligent subgrade compaction system primarily comprises vibration acceleration sensors, moisture content acquisition devices, vibratory rollers, and other auxiliary equipment. The overall hardware configuration of the novel intelligent compaction system is illustrated in Figure 5:

Equipment installation details: (1) Vibration acceleration sensors are mounted on the drum axle (30 cm from drum center); (2) Near-infrared moisture meter is fixed on the front anti-collision beam (50 cm above ground); and (3) External measuring antenna is installed on the roller roof for unobstructed GNSS signal reception.

### 3.2. Working Principle of Intelligent Vibratory Roller Real-Time Inspection System

#### 3.2.1. Intelligent Compaction System Vibration Acceleration Signal Acquisition and Calculation Principles

Intelligent compaction technology evaluates the compaction quality by analyzing the intensity of the vibratory response of the roller, inversely deriving the resistance characteristics of the fill material, and thus assessing compaction quality.

Assuming that the exciting force of the vibrating wheel follows the simple harmonic law (*Psinωt*) and ignoring the effect of gravity, the dynamic equation of the exciter is established as (2):

(2) $$M\ddot{x}=P\sin\omega t-F(x)$$

When the excitation parameters (*M, P, f*) are stable, the vibration response (e.g., acceleration) is positively correlated with the material resistance *F(x)*, and the resistance is positively correlated with the material dry density.

The linear relationship between resistance and acceleration is established through the dynamic equation as (3):

(3) $$F(x)=P\sin\omega t-M\ddot{x}-Mg$$

where *P* is the excitation force, *ω* is the frequency of the vibration circle; *t* is the vibration time; *M* is the mass of the whole vibrating body; *x* corresponds to the acceleration at a certain point; and *F(x)* represents the resistance of the compressed material to the exciter. This is further simplified as $F\left(x\right)=K\cdot a$, which shows that the resistance is directly related to the acceleration.

Field tests were conducted to determine the ratio of the subgrade resistance force to the vibratory wheel excitation force, followed by establishing the correlation between the compaction measurement value (CMV) and subgrade compaction evaluated by the differential settlement method. A vibration acceleration sensor was installed on the central shaft of the vibratory roller wheel to monitor the instantaneous acceleration influenced by the excitation force, self-gravity of the vibratory wheel, and material resistance. Through vibro-mechanical modeling and field test calibration, the vibratory wheel acceleration signal was validated to quantitatively characterize the fill material resistance and dry density.

#### 3.2.2. Principle of Real-Time Detection and Calculation of Moisture Content Data

To enable real-time subgrade moisture monitoring for determining optimal compaction procedures, a near-infrared (NIR) moisture meter was employed, leveraging the principle of near-infrared spectral absorption for precise measurement. Light exhibits both evanescent and particle properties, carrying quantifiable energy—referred to as photon energy—governed by Equation (3):

(4) e=hv

where *e* is the energy; *h* is Planck’s constant; and *v* is the frequency of light.

NIR spectroscopy offers rapid, accurate, and non-destructive detection, unaffected by environmental factors or power supply constraints, making it ideally suited to moisture content monitoring in highway subgrade construction [30].

Common near-infrared spectroscopy methods include transmission and diffuse reflection. Transmission NIR spectrometry requires the NIR beam to pass through the object, and is mainly suitable for testing homogeneous gases, transparent liquids or partially transparent solids that allow the NIR beam to pass through, but is not suitable for the rapid and accurate detection of water content in roadbeds.

**Figure 6:**
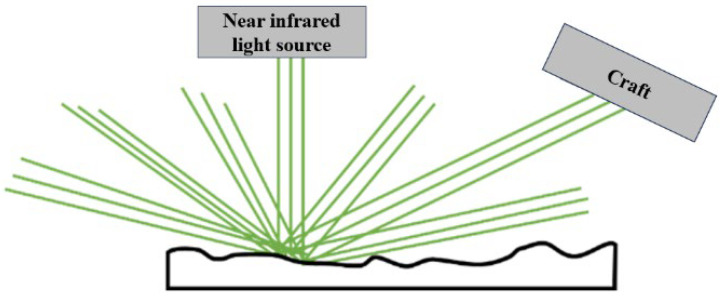
Diffuse near-infrared spectrometry measurement schematics. The green lines represents the path of diffuse light.

**Figure 7:**
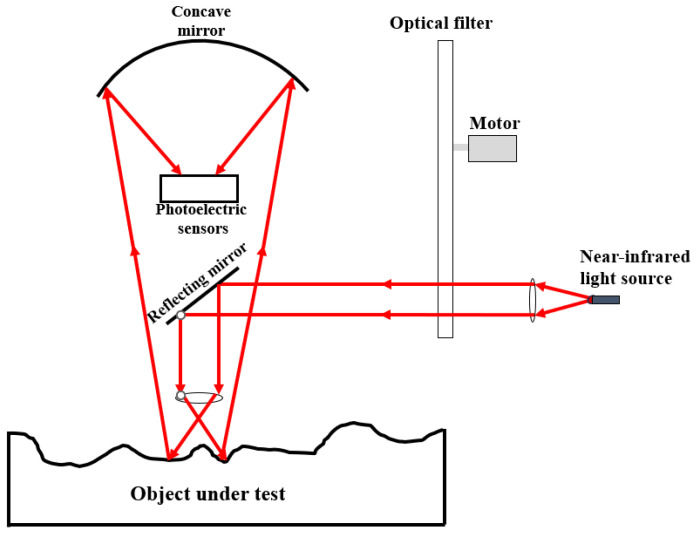
Near-infrared light moisture content measurement equipment testing principle diagram. The red arrow indicates the infrared light path.

Diffuse reflection NIR is suitable for SRM with stone content < 60%; for a higher stone content (>60%), the measurement error increases by 1.2–1.5% due to aggregate shielding. The method was calibrated against oven-drying (105 °C, 24 h) with 50 SRM samples, yielding a calibration model: Moisture = 0.98 × NIR reading + 0.3 (R^2^ = 0.96, RMSE = 0.4%). This method is suitable for testing solid particles and other objects that are not easily penetrated by near-infrared light. The specific principle is shown in Figure 6:

At present, the stability of near-infrared (NIR) moisture detection devices is primarily achieved through a dual-laser-tube indium gallium arsenide photodiode structure to test the moisture content of the measured object. The structure of the NIR moisture meter is shown in Figure 7:

Light source: 50 W halogen lamp; Detector: InGaAs photodiode (response time: 10 μs); Measurement distance: 30 ± 2 cm from soil surface to minimize interference from uneven terrain.

The site dry density *ρ_d_* (Equation (5)) was calculated from the actual moisture content measured by the NIR moisture meter Φ and the wet density *ρ*_Φ_ measured in the field, and combined with the maximum dry density obtained from the indoor tests to determine the site compaction.

(5) $$\rho_{d}=\frac{\rho_{\Phi }}{1+\Phi }$$

### 3.3. New Intelligent Compaction System Signal Acquisition, Processing and Analysis

The Python programming language is well-suited to data processing tasks, offering significant advantages in developing efficient and scalable software solutions. Consequently, the custom-developed modules of the system were implemented in Python 3.7 using Visual Studio Code 1.96.4 as the integrated development environment (IDE).

#### 3.3.1. Vibration Acceleration Signals

(a) Vibration Acceleration Signal Acquisition

**Figure 8:**
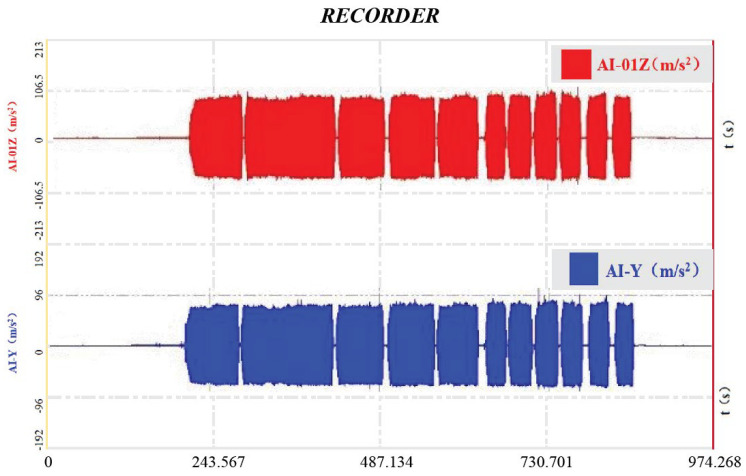
Graphical presentation of real-time monitoring of vibration acceleration signals.

The data acquisition system displays the vibration acceleration data collected by sensors in real time on the main interface of the dynamic signal acquisition and analysis system. The signals from the two vibration acceleration sensors are displayed separately, generating a real-time monitoring curve of the vibration acceleration signals to identify critical nodes in the signal acquisition process. The real-time detection curve is shown in Figure 8: red—raw time-domain vibration signal (amplitude: ±5 g); blue—FFT-processed frequency spectrum, with 2nd harmonic (50 Hz) highlighted as the key compaction index.

(b) Vibration Acceleration Signal Processing and Analysis

Vibration signals (sampled at 2000 Hz) were processed using FFT with *N* = 1024 points (frequency resolution: 1.95 Hz), focusing on the 2nd harmonic (50 Hz), which correlates most strongly with the compaction density [7]. Figure 8 shows the frequency domain spectrum, where the 2nd harmonic amplitude increases by 30% when the compaction degree rises from 90% to 96%.

(6) $$X[k]=\sum_{m=0}^{N/2-1}x[2m]\cdot e^{-j\frac{2\pi }{N/2}km}+e^{-j\frac{2\pi }{N}k}\sum_{m=0}^{N/2-1}x[2m+1]\cdot e^{-j\frac{2\pi }{N/2}km}$$

where *N* is the length of the sequence *x*[*n*]; *x*[*n*] denotes the time-domain discrete sequence; *x*[2*m*] denotes the subsequence of even terms of *x*[*n*]; *x*[2*m* + 1] is the subsequence of odd terms of *x*[*n*]; m is the index of the subsequence; and $e^{-j\frac{2\pi }{N}\cdot 2m\cdot k}$ is the complex-exponential rotation factor.

(c) Processed Vibration Acceleration Signal Output

The processed vibration acceleration signals are output in hexadecimal format with a structured data packet. The system automatically initiates data transmission upon startup, sending vibration acceleration data twice per minute. Each data packet includes a header, timestamp, output frequency, A1 peak value, A0 peak value, and a tail checksum. The entire packet uses ‘AA BB’ as the start delimiter and ‘BB AA’ as the end check code for data integrity verification.

#### 3.3.2. Real-Time Detection and Collection of Water Content of Roadbed

The system employs a near-infrared moisture meter (CM710E + HMI) produced by NDC Technologies (Dayton, OH, USA) for real-time moisture content detection. Mounted on the front anti-collision beam of the vibratory roller, the sensor ensures optimal measurement accuracy. The collected moisture data is transmitted via Ethernet, enhancing anti-interference capabilities during data transfer. The key exported parameters include the moisture acquisition time, sampling interval, frequency, instantaneous moisture content, average moisture value, and data stability metrics.

The subsequent processing of the real-time moisture data is implemented using Python code.

#### 3.3.3. Acquisition, Processing and Synthesis of Positioning Signals

During vibratory compaction operations, the external measuring antenna and SKY2 positioning receiver continuously acquire navigation satellite data. Leveraging the XCOM serial debugging assistant, this positioning data is transmitted to the computer via serial communication protocols.

**Figure 9:**
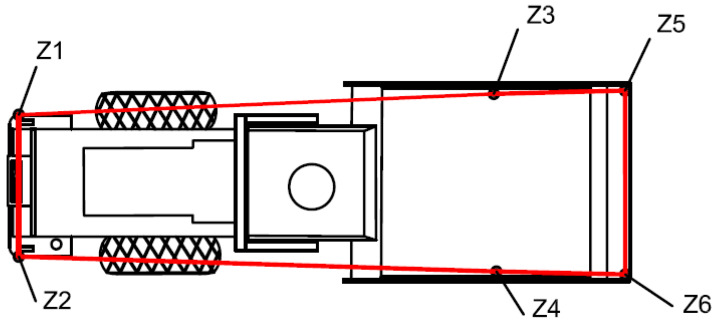
Vibratory roller outline. The red line denotes the outline of the roller marked with six points.

Six reference points (Z1–Z6) are defined relative to the drum center: Z1/Z2 (left/right drum edges, 1.05 m from center), Z3/Z4 (front/rear of drum, 0.5 m from center), and Z5/Z6 (roller cab corners). Their coordinates are recorded at 10 Hz to generate the roller contour with a spatial resolution of 0.1 m. Positioning data (GNSS timestamp) and vibration data (sensor timestamp) are synchronized using a hardware trigger, ensuring time deviation <10 ms. This enables the accurate mapping of CMVs to their spatial locations. The schematic layout is shown in Figure 9. A Python-based real-time trajectory visualization module generates the compaction path, dynamically adjusting the width of the operation track line to monitor under-compacted or missed areas.

## 4. Intelligent Compaction Monitoring of the Whole Process of Mixed Soil and Stone Roadbeds

This study takes the K70+45–K70+245 section of the fourth bid of the Linlin (Linzi-Linyi) Expressway as the field engineering application case for the intelligent compaction technology of soil–rock mixed-fill subgrades. The design speed is 120 km/h, the subgrade width is 34.5 m, and the traffic load is classified as heavy traffic.

The subgrade filling is implemented by layered horizontal filling, with the loose fill layer thickness controlled at 40 cm. The stone material has a strength of no less than 15 MPa, predominantly granite, and the uniaxial ultimate compressive strength of the rock is 45–52 MPa. The fill material has an unevenness coefficient of 15–20, a stone content of 30%, an optimum moisture content of 8%, and a construction moisture content range of 7–10%. A vibratory roller equipped with an intelligent compaction system was used for rolling on the test section, with six passes performed.

### 4.1. Intelligent Compaction System Quality Testing

#### 4.1.1. Detection of Roadbed Compaction Quality by Settlement Difference Method

**Figure 10:**
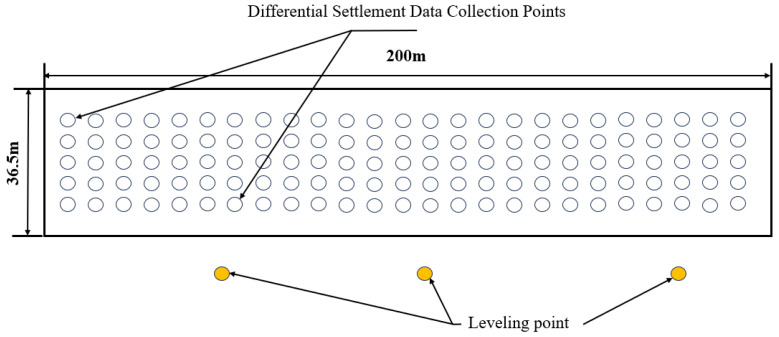
Distribution of settlement collection points and location of leveling devices.

The settlement difference method was employed to monitor the entire intelligent compaction process. After each compaction pass, 50 measurement points were selected in a 5 m × 5 m grid (covering the entire 34.5 m-wide subgrade, including five points at each edge) for settlement detection. The distribution of these points (labeled S1–S50) and the position of the digital level (Leica DNA03, accuracy ± 0.3 mm) are illustrated in Figure 10.

**Figure 11:**
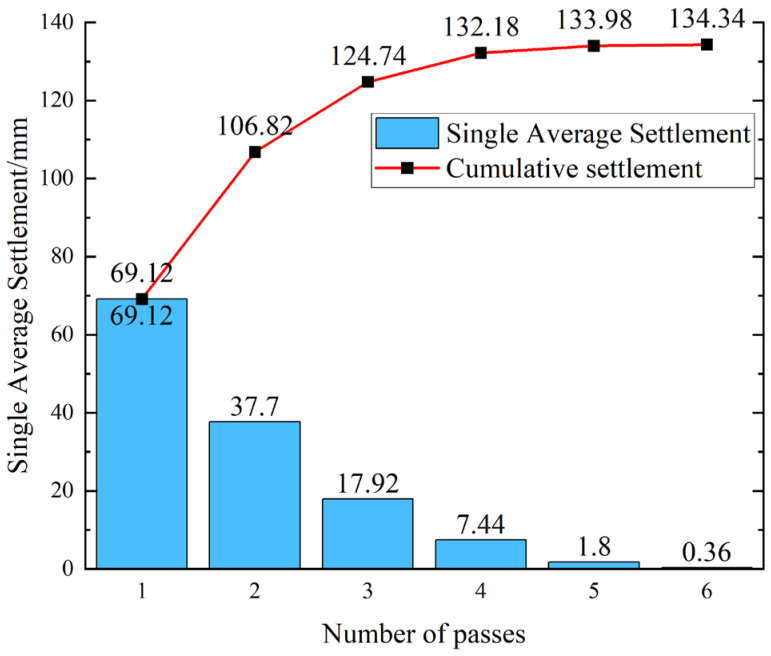
Single average settlement vs. cumulative settlement.

As shown in Figure 11, field differential settlement measurements indicate that the soil–rock mixed subgrade is deemed compacted (qualified) when the differential settlement is less than 2 mm, the average cumulative settlement curve of measuring points tends to be linear with minimal increase, the compaction differential settlement does not exceed 2 mm, and the standard deviation is less than 1 mm.

Combined with cumulative settlement data from numerous other measuring points, for the soil–rock mixed subgrade with a loose thickness of 40 cm and six passes of rolling, the maximum cumulative settlement is 141 mm, the minimum is 125 mm, and the average is 134.52 mm. The compaction quality of the soil–rock mixed subgrade meets the specification requirements.

#### 4.1.2. Intelligent Compaction Monitoring of Mixed Soil and Gravel Roadbeds in the Whole Process

**Figure 12:**
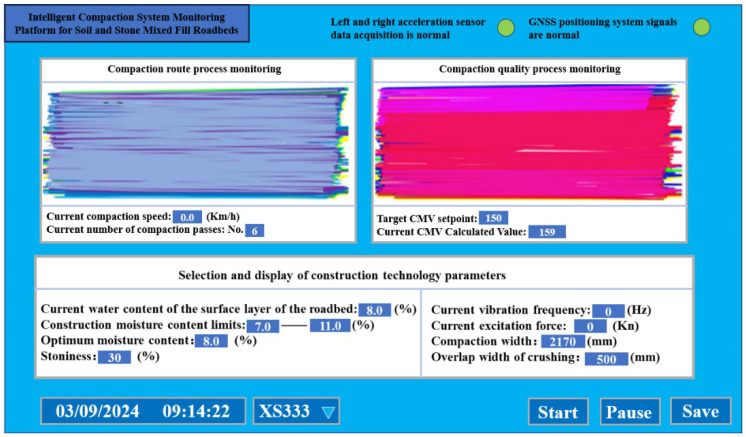
Intelligent compaction system visualization and monitoring.

The vibratory roller equipped with the intelligent compaction system for the soil–rock mixed-fill subgrade was used to crush the surface of the K70+45–70+245 section based on the crushing construction technical parameters determined in the previous test section. Figure 12. Visualization of intelligent compaction monitoring results, including the (a) compaction trajectory (color-coded by pass number: 1st pass yellow, 6th pass gray); (b) CMV distribution under-compacted zones (yellow/blue) were re-compacted with 1–2 additional passes, resulting in 100% compliance.

In the rolling trajectory diagram, the driving path of the vibratory roller on the compacted surface is visualized, with color-coded zones indicating potential missed areas for immediate identification. This allows for targeted re-compaction in subsequent construction phases to address any coverage gaps.

The compaction effect diagram provides a real-time visual assessment of compaction quality, highlighting under-compacted zones with distinct markers. This enables the vibratory roller to promptly rectify weak compaction areas through supplementary rolling, ensuring uniform density across the entire construction surface.

Additionally, the near-infrared moisture meter continuously measures the subgrade moisture content, with real-time data displayed on the system interface. This facilitates the instant detection of moisture levels exceeding the specified range (7–10%), enabling proactive adjustments to maintain optimal construction conditions.

### 4.2. Analysis of Intelligent Compaction Technology for Soil and Stone Mixed Fill Road Base

#### 4.2.1. Quality Control During Construction

The intelligent compaction system for soil–rock mixed-fill subgrades innovatively establishes a compaction quantification model for multi-phase media, breaking through the limitation that traditional intelligent compaction technologies are inapplicable to non-homogeneous materials. The full-section compaction uniformity control model enables the real-time graphic warning of under-compaction and over-compaction conditions; among 500 monitored points, 488 were correctly identified, yielding a 97.6% recognition rate. It also provides real-time display of the Compaction Measurement Value (CMV) and mechanical kinematic parameters, allowing drivers to dynamically adjust the vibration mode and enabling construction managers to accurately optimize the combination of compaction process parameters. Engineering practice has shown that this technology increases the compaction qualification rate of soil–rock mixed-fill subgrades to 98%.

#### 4.2.2. Quality Traceability of Milling Operations

Currently, the quality control of intelligent compaction operations for subgrades still widely faces the technical challenges of high manual intervention and strong reliance on discrete detection. Aiming at the engineering characteristics of soil–rock mixed-fill subgrades, this intelligent compaction system innovatively constructs an online compaction quality diagnosis system by integrating near-infrared spectrometers and moisture sensors, enabling synchronous monitoring of the compaction excitation force field distribution, mechanical movement trajectory, and water content status of the filler material.

At the data application layer, after feature extraction and fast Fourier transform (FFT) processing via Python code, the system generates digital compaction quality process control maps containing compaction cloud maps, superimposed mechanical trajectories, and process parameter time–history curves. These maps allow precise traceability to specific compaction wheel tracks, providing data-chain evidence with engineering legal validity for quality defect tracing. Engineering practice has verified that this technological system reduces the manpower input for quality inspection by 65% compared to traditional methods.

#### 4.2.3. Accelerating Construction Progress

Through the fusion and analysis of multi-source sensing data, the intelligent compaction system for soil–rock mixed-fill subgrades enables the real-time dynamic monitoring of process parameters and mechanical response characteristics of the compacted surface. The ‘monitoring–analysis–execution’ cycle (15 min per loop) shortened the construction period of 200 m subgrade from 20 days (traditional) to 14 days (30% reduction). Cost reduction (18–22%) includes 25% less rework (from 10% to 2.5% of total length), 30% lower labor costs, and 15% less machinery idle time (verified via 3 months of project records).

## 5. Conclusions

This study systematically reviews the research progress in compaction quality control theories and intelligent compaction technologies for soil–rock mixed-fill subgrades at home and abroad. Based on the technical key points of soil–rock mixed-fill subgrade construction, an embedded computing platform based on Python is developed using vibratory acceleration sensors, positioning signal receivers, near-infrared moisture meters, and other related equipment to realize the fusion processing of heterogeneous data from multiple sources in the vibration frequency domain, spatial trajectory, and moisture content. Through a layered architecture design, a whole-process digital solution for fusion visualization is constructed, which has been verified by engineering to achieve a compaction uniformity determination accuracy of ±2.5 kN/m^3^.

Through extensive on-site inspection data, the application scope and inspection accuracy of intelligent compaction technology are expanded. Meanwhile, the engineering feasibility of the intelligent compaction system is validated by integrating the construction practice of the Linlin Expressway.

(a) Field materials from Linlin Expressway show a soil–stone ratio of 30:70 (range: 28:72 to 32:68, *n* = 4 sampling points), consistent with laboratory preparation. The four soil samples exhibit poor gradation (curvature coefficients Cc = 0.62–0.94 < 1) as per JTG 3430-2020. Indoor compaction tests yielded a maximum dry density (MDD) of 2.15 ± 0.05 g/cm^3^ and optimum moisture content (OMC) of 8.0 ± 0.5% (mean ± SD), which were used to calibrate the intelligent system’s compaction indices (e.g., CMV threshold for 96% compaction degree).
(b) A critical limitation of conventional Intelligent Compaction Technology (ICT) lies in its neglect of moisture content—a factor that directly impacts compaction quality. To address this gap, the present study introduces an innovative integration of moisture content monitoring into intelligent vibratory rollers, resulting in a novel hardware–software system with three pivotal technical advancements, each targeting specific challenges in traditional ICT: (1) Real-time moisture content correction based on Near-Infrared (NIR) spectroscopy. This advancement addresses the technical challenge of inaccurate, off-line moisture measurement (e.g., time-consuming oven-drying methods). By leveraging NIR spectroscopy, the system achieves real-time moisture quantification with a high coefficient of determination (R^2^ = 0.96) when validated against the gold-standard oven-drying method, eliminating the lag and error associated with traditional off-line testing. (2) FFT-based Compactness Meter Value (CMV) harmonic analysis Conventional CMV analysis often struggles to extract stable, reliable indicators for compaction quality evaluation. To resolve this, the study proposes focusing on the 2nd harmonic of FFT-processed CMV data—an innovation that enhances the robustness of compaction stability assessment by isolating a harmonic component with strong correlation to uniform compaction, avoiding interference from random noise in raw CMV signals. (3) Python-enabled time-synchronized data fusion. Multi-source data (e.g., moisture, CMV, location) in traditional ICT systems frequently suffer from timestamp misalignment, which undermines the integrity of integrated analysis. This study overcomes this technical barrier through a Python-based fusion framework, achieving a timestamp alignment accuracy of <10 ms. This ensures temporal consistency across all sensing modules, a prerequisite for accurate cross-parameter correlation analysis. Composed of dedicated sensors and edge computing units, the integrated hardware-software system directly addresses the aforementioned technical challenges of traditional ICT. Validation using over 500 field data points collected from real-world construction scenarios demonstrates that the system reduces the measurement error by 40% compared to conventional ICT solutions, confirming its practical applicability and technical superiority.
(c) Field application in the Linlin Expressway test section showed that the system outperforms the traditional settlement difference method: the compaction qualification rate increased from 82% to 98%, and the anomaly detection accuracy reached 97.6% (*n* = 200 test points). The system exhibited good stability during 30 days of continuous operation, with real-time reminders for under-compacted zones (response time < 5 s) effectively reducing rework rates. A comparison of this system with traditional ICT is shown in Table 2.

## Figures and Tables

**Table 1:** Gradation indicators at different locations.

Location Number	d_10_/mm	d_30_/mm	d_60_/mm	Inhomogeneity Factor *C*_u_	Curvature Coefficient	Location Number
W-1	0.31	1.67	9.6	30.97	0.94	undesirable
W-2	0.34	1.73	9.8	28.82	0.89	undesirable
W-3	0.29	1.64	11.7	40.34	0.79	undesirable
W-4	0.33	1.42	9.8	29.76	0.62	undesirable

**Table 2:** Comparison of metrics improvement between traditional ITC and this system.

Norm	Traditional ICT	New System	Enhancement
Moisture Response Delay	>5 min	<10 s	97%
CMV calibration accuracy	±15%	±6%	60%
Resolution of compaction defects	>0.5 m^2^	<0.1 m^2^	80%

## Data Availability

The original contributions presented in this study are included in the article. Further inquiries can be directed to the corresponding author.
